# Efficacy of SGLT2 inhibitors in IgA nephropathy associated with alcoholic liver cirrhosis accompanied by nephrotic syndrome: a case report

**DOI:** 10.3389/fneph.2023.1331757

**Published:** 2024-01-22

**Authors:** Yusuke Yoshimura, Daisuke Ikuma, Hiroki Mizuno, Kei Kono, Keiichi Kinowaki, Hisashi Sugimoto, Hisashi Kamido, Yuichiro Sawada, Masato Mizuta, Shigekazu Kurihara, Yuki Oba, Masayuki Yamanouchi, Tatsuya Suwabe, Kenichi Ohashi, Yoshifumi Ubara, Naoki Sawa

**Affiliations:** ^1^ Nephrology Center, Toranomon Hospital Kajigaya, Kawasaki, Kanagawa, Japan; ^2^ Department of Pathology, Toranomon Hospital, Minato-ku, Tokyo, Japan

**Keywords:** IgA nephropathy, alcoholic liver cirrhosis, kidney biopsy, SGLT2 inhibitor, nephrotic syndrome

## Abstract

We present a 51-year-old male patient with a history of Child-Pugh Grade B alcoholic liver cirrhosis (ALC) who developed renal impairment (serum creatinine of 2.00 mg/dL) and nephrotic syndrome (a urinary protein level of 4.35 g/gCr). The patient was diagnosed with immunoglobulin A nephropathy (IgAN) associated with ALC based on findings from comprehensive evaluations, including markedly elevated serum IgA levels (883.7 mg/dL), a kidney biopsy revealing significant IgA deposition in the para-mesangial area, and a liver diagnosis showing long-standing advanced ALC. Our treatment approach involved initiating dapagliflozin therapy, a sodium-glucose cotransporter-2 (SGLT2) inhibitor, alongside strict alcohol abstinence. Remarkably, the patient demonstrated a dramatic reduction in proteinuria within one week of dapagliflozin administration. No hypoglycemic events were observed. This case adds valuable clinical insights into the potential therapeutic role of SGLT2 inhibitors in IgAN associated with ALC. Specifically, in cases where conventional steroid therapies may be contraindicated due to coexisting comorbidities such as diabetes or obesity, dapagliflozin emerges as a potentially efficacious alternative. Further investigations are warranted to validate these preliminary observations.

## Case report

The patient was a 51-year-old male with a 27-year history of daily alcohol consumption consisting of 500 mL of beer and 1,400 mL of shochu with water. According to his medical history, at age 44, he was diagnosed with a pituitary adenoma and underwent transsphenoidal surgery. Subsequently, he developed postoperative growth hormone deficiency.

At age 45, he was diagnosed with alcoholic liver disease and hepatic diabetes. Due to an elevated hemoglobin A1c (HbA1c) level of 10%, sitagliptin therapy was initiated when he was 47 years old. However, at age 49, his blood glucose levels improved dramatically, possibly due to a decrease in glucogenesis resulting from reduced liver function. Consequently, he maintained an HbA1c level in the 5% range without medication. His diabetes was not complicated by either neuropathy or retinopathy. He was diagnosed with hypertension, dyslipidemia, and hyperuricemia at the age of 50 years.

His primary care physician observed bilateral lower leg edema, hematuria, and proteinuria, which subsequently led to his admission to our institution for further evaluation.

His medication regimen at admission included azilsartan 10 mg, nifedipine CR 20 mg, and topiroxostat 80 mg.

Upon admission, the patient had a height of 180 cm and a weight of 89.1 kg, yielding a body mass index (BMI) of 27.5 kg/m^2^. He was conscious and alert, and his blood pressure was 114/76 mmHg. The physical examination showed mild pitting edema in the bilateral lower legs. The results of the blood examination were as follows: total protein, 7.4 g/dL; albumin, 2.9 g/dL; aspartate aminotransferase, 42 U/L; alanine aminotransferase, 32 U/L; total bilirubin, 0.9 mg/dL; creatinine, 2.00 mg/dL; estimated glomerular filtration rate, 29.4 mL/min/1.73 m^2^; urea nitrogen, 36 mg/dL; immunoglobulin A (IgA), 883.7 mg/dL; HbA1c, 5.6%; hemoglobin, 15.5 g/dL; platelet count, 114 x 10^3^/µL; prothrombin time, 99.6%; hepatitis B surface antigen, negative; hepatitis B surface antibody, negative; and hepatitis C antibody, negative. Urinalysis revealed a urinary red blood cell count of 10–19/high power field and a urinary protein level of 4.35 g/gCr. Abdominal ultrasonography revealed a heterogeneous liver echotexture, nodular and dull-edged liver surface, splenomegaly, and slight ascites. The transient elastography assessment, conducted using FibroScan^®^ (Echosens, Paris, France), indicated a liver stiffness measurement of 38.3 kPa (reference range 2–7 kPa), which is consistent with grade F4 liver fibrosis according to the new Inuyama classification. Gastrointestinal endoscopy revealed esophageal varices with a red color sign. Based on these results, a diagnosis of liver cirrhosis (Child-Pugh grade B) with portal hypertension was made.

To investigate the pathogenesis of renal dysfunction with severe proteinuria, a kidney biopsy was conducted.

## Kidney biopsy findings

**Figure 1:**
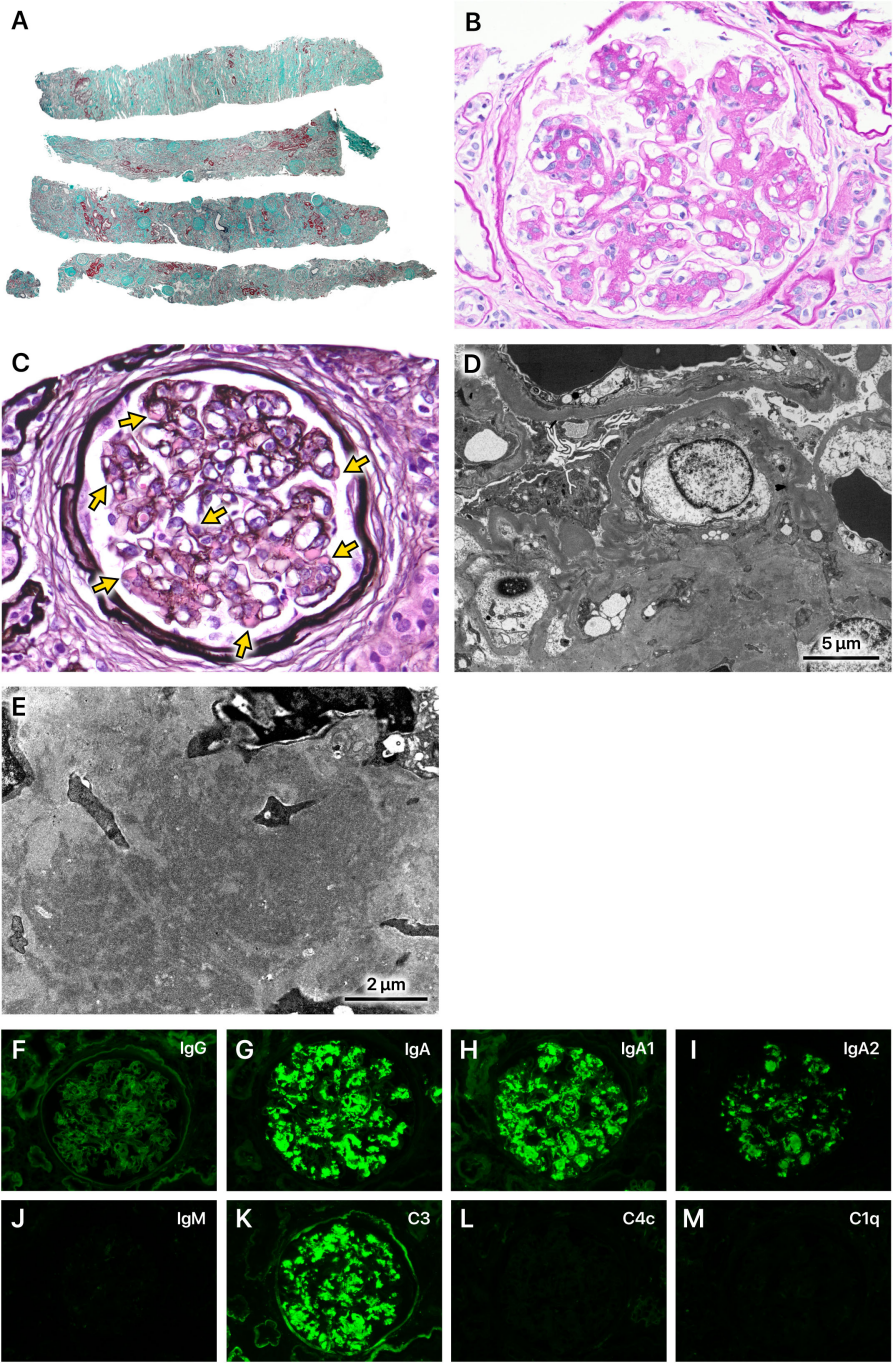
Kidney biopsy findings. **(A)** A total of 45 glomeruli were observed. Of these, 29 were sclerotic and classified as S1 according to the Oxford Classification of IgA nephropathy (2016). The tubulointerstitial areas displayed diffuse marked fibrosis (T2). Masson-trichrome staining, x40. **(B)** Mesangial cells increased in many glomeruli (M1). Intravascular cell proliferation was absent (E0). No crescents were observed (C0). Preserved glomeruli were slightly enlarged. The mesangial cells showed cleaved fission images. Periodic acid-Schiff staining, x400. **(C)** The mesangial matrix was mildly increased. Large hemispherical deposits of para-mesangial regions were also observed (arrows). Periodic acid methenamine silver staining, x400. **(D, E)** Electron microscopy revealed very large electron-dense deposits in the subendothelial and mesangial areas. **(F–M)** Immunofluorescence staining showed IgA1-dominant deposition in the mesangial region, which was characterized by a very large amount of IgA deposition.

The kidney biopsy showed a cortex to medulla ratio of 9:1. A total of 45 glomeruli were observed, 29 of which were sclerotic. The tubulointerstitial areas displayed diffuse marked fibrosis (**Figure 1A**). Periodic acid-Schiff staining showed increased mesangial cells in many glomeruli. Intravascular cell proliferation was absent. No crescents were observed. Preserved glomeruli were slightly enlarged. The mesangial cells showed cleaved fission images (**Figure 1B**). Periodic acid methenamine silver (PAM) staining showed a mild increase in the mesangial matrix. Large hemispherical deposits of para-mesangial regions were also observed (**Figure 1C**). Electron microscopy revealed large electron-dense deposits (EDD) in the subendothelial and mesangial areas (**Figures 1D, E**). Fluorescent immunostaining showed IgA1-dominant IgA deposition in the mesangial region, which was characterized by a very large amount of IgA deposition (**Figures 1F–M**). Based on these observations, the patient was pathologically diagnosed with IgA nephropathy (IgAN), classified as M1, E0, S1, T2, and C0 according to the Oxford Classification of IgA nephropathy (2016) (1).

The patient had a long history of excessive alcohol consumption and exhibited advanced alcoholic liver cirrhosis (ALC). Serum IgA levels were markedly elevated compared to those typically observed in primary IgAN. Furthermore, a kidney biopsy revealed diffuse and severe interstitial fibrosis, substantial deposits in the para-mesangial region as revealed by PAM staining, extensive EDD in the subendothelial and mesangial regions on electron microscopy, and significant IgA deposits on immunofluorescence staining. These findings all indicated ALC as a potential etiological factor for IgAN.

## Clinical course

**Figure 2:**
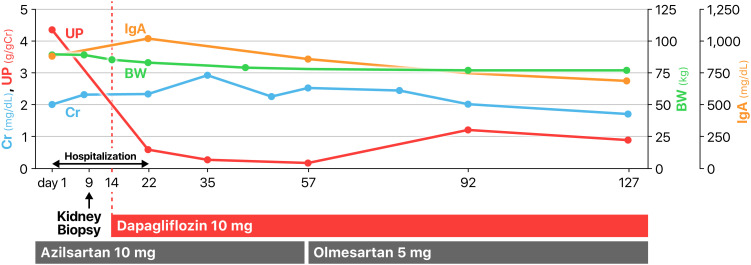
Clinical course. BW, body weight; Cr, creatinine; UP, urinary protein; IgA, immunoglobulin A.

**Figure 2** outlines the patient’s clinical course following admission. Based on the diagnosis of IgAN associated with ALC (IgAN-ALC), alcohol consumption was strictly prohibited. Furthermore, dapagliflozin therapy was initiated at a dosage of 10 mg/day, anticipating its renoprotective benefits while conducting vigilant monitoring for potential hypoglycemic events. Approximately one week after the commencement of dapagliflozin treatment, the patient’s urinary protein levels markedly reduced to below 1 g/gCr, and hematuria disappeared. Concurrently, his body weight decreased by over 10 kg. The patient was discharged on the 22nd day and the outpatient monitoring was continued. The patient continued to take azilsartan, a medication prescribed previously, and his blood pressure remained largely within the optimal range throughout the course of treatment. He adhered to the alcohol prohibition and maintained urinary protein levels consistently below 1 g/gCr, with a stable serum creatinine level at 2–3 mg/dL. The serum IgA level also decreased to 687 mg/dL approximately 4 months after initiating dapagliflozin therapy. The patient did not experience hypoglycemia during the entire clinical course.

## Discussion

We present a case of IgAN-ALC, with a marked decrease in proteinuria after administration of dapagliflozin, a sodium-glucose cotransporter-2 (SGLT2) inhibitor.

Newell GC et al. reported that 50–100% of patients with ALC exhibit glomerular injury and 30–90% show IgA deposits in the mesangial region (2).

Normally, serum IgA is recognized by asialoglycoprotein receptors (ASGP-Rs) on the surface of hepatocytes and is processed within hepatocytes (3). However, in liver cirrhosis patients, the number of hepatocytes decreases and the polarity of hepatocytes is lost. This leads to the impaired uptake and clearance of serum IgA and IgA immune complexes. Secretory IgA produced by plasma cells in the lamina propria of the intestinal mucosa usually reaches the liver via the portal vein and is excreted into bile through hepatocyte ASGP-R. However, in liver cirrhosis patients, the enterohepatic circulation is impaired. Due to portal hypertension and the resulting portosystemic shunt, some IgA immune complexes in the blood bypass the liver and directly enter the renal glomeruli (4). Furthermore, in heavy alcohol drinkers, exposure to food antigens and gut bacteria due to chronically damaged intestinal mucosa by alcohol can lead to increased production of IgA antibodies (5, 6). These mechanisms contribute to the elevated risk of IgAN in patients with ALC.

It has been reported that no histologic feature distinguishes IgAN-ALC from primary IgAN (7). However, both in this case and in previously reported cases of IgAN-ALC, characteristic findings, such as substantial deposits in the para-mesangial region on PAM staining, extensive EDD in the subendothelial and mesangial regions on electron microscopy, and significant IgA deposits on immunofluorescence staining, were observed compared to primary IgAN (8). Higher serum IgA levels in blood examination, which are common in liver cirrhosis patients (9), also support the possibility of hepatic IgAN. These findings may be attributed to the possibility that a greater amount of IgA immune complexes flow into the glomeruli in IgAN-ALC than in primary IgAN due to the mechanisms described above.

Although there is still no established treatment for IgAN-ALC (7), Takada et al. reported two cases in which hematuria, proteinuria, and a large EDD observed by electron microscopy successfully disappeared following alcohol abstinence and methylprednisolone pulse therapy (8). Given that IgAN-ALC involves the formation of IgA immune complexes, similar to primary IgAN, it would be logical to assume that high-dose steroid therapy, the standard treatment for primary IgAN, would also be efficacious in treating IgAN-ALC.

However, IgAN-ALC presents a distinct therapeutic challenge. According to Kidney Disease: Improving Global Outcomes (KDIGO) guidelines, in patients with IgAN who also have liver cirrhosis, therapy with glucocorticoids should be approached with extreme caution or avoided entirely (10). A total of 32.5% of patients with ALC have diabetes mellitus and 38.8% are obese (BMI >25 kg/m^2^) (11). Thus, the use of steroids can be problematic. In this case, we opted against steroid therapy mainly due to the patient’s concurrent diabetes mellitus and obesity. Given these comorbidities and a pretreatment eGFR exceeding 25 mL/min/1.73 m^2^, we selected an SGLT2 inhibitor as the preferred treatment approach.

The DAPA-CKD study demonstrated that dapagliflozin significantly reduced the risk of a composite of a sustained decline in the eGFR of at least 50%, end-stage kidney disease, or death from renal or cardiovascular causes in chronic kidney disease (CKD) patients with an eGFR of 25–75 mL/min/1.73 m^2^ (12). Further analysis limited to 270 IgAN patients in the DAPA-CKD study also indicated that dapagliflozin was effective in reducing the frequency of renal impairment, slowing the decline in eGFR, and reducing proteinuria with a favorable safety profile (13).

Among antihyperglycemic agents, SGLT2 inhibitors are recognized for their minimal risk of hypoglycemia, and metformin and dipeptidyl peptidase-4 inhibitors are recommended for patients with impaired liver function (14). In the present case, we suspected a reduction in gluconeogenesis and initiated dapagliflozin therapy during hospitalization, paying close attention to the risk of hypoglycemia. Throughout the in-hospital stay and subsequent outpatient follow-up, the patient experienced no episodes of hypoglycemia, indicating excellent tolerability.

SGLT2 inhibitors have also been reported to offer extraglycemic benefits. In patients with CKD complicated by diabetic kidney disease, as in this case, SGLT2 inhibitors exert renal protective effects by acting on both the glomeruli and the tubulointerstitial regions. In the glomeruli, SGLT2 inhibitors reduce intraglomerular pressure, improve hyperfiltration, and decrease proteinuria. This effect on lowering intraglomerular pressure is also a feature of the angiotensin receptor blockers the patient was already taking; however, the mechanisms of intraglomerular pressure reduction by renin-angiotensin system (RAS) inhibitors and SGLT2 inhibitors differ. While RAS inhibitors lower intraglomerular pressure by dilating the efferent arterioles, SGLT2 inhibitors normalize the abnormally dilated afferent arteriole diameter, thus reducing intraglomerular pressure (15). Additionally, in the tubulointerstitial region, SGLT2 inhibitors are known to normalize tubuloglomerular feedback by inhibiting sodium reabsorption. Furthermore, mechanisms for renal protection mediated by metabolic regulation, such as normalization of the glycolytic pathway and the citric acid cycle (16) and elevation of blood ketone body concentration to assist adenosine triphosphate production (17), have also been reported. In a nationwide cohort study, Bea et al. observed that the use of SGLT2 inhibitors was linked to a decreased risk of major hepatic events, compensated liver cirrhosis, hepatic decompensation events, and all-cause mortality compared to the use of dipeptidyl peptidase-4 inhibitors in patients with type 2 diabetes (18). Our search did not uncover comprehensive reports detailing the long-term clinical course of IgAN-ALC or hepatic IgAN. This evidence gap highlights the need for future investigations to better understand the long-term outcomes of these conditions.

In this case, a marked reduction in proteinuria and body weight followed the initiation of an SGLT2 inhibitor and alcohol abstinence. This improvement may be attributed to the combined effects of the SGLT2 inhibitor and alcohol cessation, which lowered afferent arteriolar pressure and corrected fluid overload, thereby contributing to reduced glomerular hyperfiltration. Additionally, serum IgA levels also significantly decreased, suggesting that continued abstinence from alcohol may have led to a reduction in the production of IgA and IgA immune complexes. Furthermore, Noah et al. showed that empagliflozin ameliorated liver fibrosis and reduced portal hypertension through the inhibition of the galactin-1/neuropilin-1 signaling pathways in an experimental liver fibrosis rat model (19). The amelioration of portal hypertension may also potentially contribute to a reduction in IgA levels associated with improved hepatic circulation and subsequently to a decrease in proteinuria.

This case suggests that SGLT2 inhibitors may be useful for renal protection in IgAN-ALC, similar to primary IgAN. Further accumulation of cases is warranted to validate this hypothesis.

## Data availability statement

The original contributions presented in the study are included in the article. Further inquiries can be directed to the corresponding author.

## Ethics statement

Written informed consent was obtained from the individual for the publication of this article.

## Author contributions

YY: Conceptualization, Data curation, Formal analysis, Investigation, Methodology, Software, Visualization, Writing – original draft, Writing – review & editing. DI: Project administration, Writing – review & editing. HM: Writing – review & editing. KKo: Data curation, Writing – review & editing. KKi: Data curation, Writing – review & editing. HS: Writing – review & editing. HK: Writing – review & editing. YS: Writing – review & editing. MM: Writing – review & editing. SK: Writing – review & editing. YO: Writing – review & editing. MY: Writing – review & editing. TS: Writing – review & editing. KO: Data curation, Writing – review & editing. YU: Supervision, Writing – review & editing. NS: Conceptualization, Investigation, Project administration, Supervision, Validation, Writing – original draft, Writing – review & editing.
